# Inhibition of Glucose-6-Phosphate Dehydrogenase Reverses Cisplatin Resistance in Lung Cancer Cells via the Redox System

**DOI:** 10.3389/fphar.2018.00043

**Published:** 2018-01-31

**Authors:** Weipeng Hong, Peiheng Cai, Chuncao Xu, Di Cao, Weibang Yu, Zhongxiang Zhao, Min Huang, Jing Jin

**Affiliations:** ^1^School of Pharmaceutical Sciences, Sun Yat-sen University, Guangzhou, China; ^2^School of Chinese Materia Medica, Guangzhou University of Chinese Medicine, Guangzhou, China

**Keywords:** pentose phosphate pathway, G6PD, cisplatin resistance, A549, A549/DDP, redox

## Abstract

The pentose phosphate pathway (PPP), which branches from glycolysis, is correlated with cancer cell proliferation, survival and senescence. In this study, differences in the metabolic profile of the PPP and the redox status of human lung carcinoma A549 cells and cisplatin-induced multidrug-resistant A549/DDP cells were analyzed and evaluated. The results showed that A549/DDP cells exhibited differential PPP-derived metabolic features and redox-related molecules. A549/DDP cells exhibited increased expression and enzymatic activity of PPP enzyme glucose-6-phosphate dehydrogenase (G6PD). Furthermore, as demonstrated by the apoptotic rate, cell viability, and colony formation, inhibition of G6PD by siRNA or an inhibitor sensitized A549/DDP cells to cisplatin. Additionally, inhibition of G6PD restored the cisplatin sensitivity of A549/DDP cells by influencing redox homeostasis. In conclusion, overcoming cisplatin resistance through inhibition of G6PD could improve the understanding of the mechanisms underlying cisplatin-induced resistance in human lung cancer and may provide insights into the therapeutic potential of this treatment to combat resistance.

## Introduction

Annually, approximately 1.5 million new cancer cases are diagnosed as lung cancer worldwide, and 85% of them are non-small-cell lung cancer (NSCLC) (Ardizzoni et al., 2007; Le, 2010). Cisplatin-based chemotherapy is a commonly used chemotherapy regimen in advanced NSCLC patients (Gridelli et al., 2005). However, one of the main factors contributing to the failure of this chemotherapy regimen is the development of drug resistance, which affects the prognosis and survival of the patients (Siddik, 2003). Therefore, a better understanding of the mechanisms of cisplatin-induced resistance and finding potential therapeutic strategies to combat it are of great significance. Metabolic reprogramming has been increasingly shown to be an obvious feature in most cancers, and the metabolic pathway has been closely associated with potential efficient treatments or drug sensitivity enhancements in cancer (Zhao et al., 2013). The pentose phosphate pathway (PPP), a branch of glycolysis, as the first committed step of glucose metabolism, was recently demonstrated as a vital factor in cancer growth and survival (Jiang et al., 2014). PPP is composed of the following phases: the oxidative branch and the non-oxidative branch. The oxidative branch is a major source of reduced nicotinamide adenine dinucleotide phosphate (NADPH) and ribulose-5-phosphate (Ru5P), which plays a crucial role in combating oxidative stress and provides the backbone for the synthesis of ribonucleotides. The non-oxidative branch is composed of a series of reversible reactions and generates several important metabolites, such as fructose-6-phosphate (F6P), glyceraldehyde-3-phosphate (G3P) and pentose phosphates, which provide supplements for glycolysis and anabolic demands (Patra and Hay, 2014).

Due to the abnormal demands of proliferation, the phenomenon of a hyperactive PPP usually manifests itself in many cancers, such as lung cancer, liver cancer, pancreas cancer and leukemia (Ying et al., 2010; Lin et al., 2015; Kowalik et al., 2017; Elf et al., 2017), and the oxidative branch plays a predominant part, since it regulates redox homeostasis by generating NADPH. NADPH is a common molecule required in the biosynthesis of fatty acids and cholesterol and the generation of reduced glutathione (GSH). Both GSH and NADPH are essential for intracellular redox homeostasis. Numerous data suggest that, caused by a high proliferative advantage, the redox status of most cancer cells usually differs from that of normal cells and thus promotes tumor progression and development and influences cancer therapy (Gorrini et al., 2013; Nogueira and Hay, 2013). In response to ROS accumulation caused by some chemotherapies, cancer cells commonly activate PPP to alleviate oxidative stress, especially the oxidative branch, which gradually obtain drug resistance (Patra and Hay, 2014). Therefore, the ability to regulate the redox status by the oxidative branch of the PPP is increasingly associated with cancer chemotherapy.

In this study, we aimed to find an efficient approach to overcome the cisplatin-induced resistance of lung cancer cells. By targeting metabolomics, we found differences in the PPP between cisplatin-induced multidrug-resistant A549/DDP cells and their parental A549 cells, and A549/DDP cells also exhibited a more predominant redox system. Based on this, we revealed the role Glucose-6-phosphate dehydrogenase (G6PD) in cisplatin-induced resistance in A549/DDP cells. G6PD is the first rate-limiting enzyme of the oxidative branch, which simultaneously generates the first molecule of NADPH. Cancer cells often express relatively high levels of G6PD to match the needs of rapid proliferation. In this study, we found that inhibition of G6PD could effectively restore the cisplatin sensitivity of A549/DDP cells.

## Materials and Methods

### Chemicals and Reagents

6-Aminonicotinamide (6-AN) and *N*-Acetyl-L-cysteine (NAC) were purchased from Sigma-Aldrich (St. Louis, MO, United States). *Cis*-diamminedichloroplatinum (II) (cisplatin, CDDP) was purchased from Tokyo Chemical Industry (Tokyo, Japan). Dimethyl sulfoxide (DMSO) and 3-(4,5-dimethyl-2-thiazolyl)-2,5-diphenyl-2-H-tetrazolium bromide (MTT) were purchased from MP Biomedicals (Santa Ana, CA, United States). Rabbit polyclonal anti-G6PD (8866), anti-PGD (13389), anti-TKT (8616) and anti-GAPDH (8884) were purchased from Cell Signaling Technology (Danvers, MA, United States), Rabbit polyclonal anti-TALDO1 (D123398) was purchased from Sangon Biotech Co., Ltd. (Shanghai, China). Other chemicals were of analytical grade from commercial suppliers.

### Cell Culture and Transient Transfection Assays

A549 and A549/DDP cells were derived from a cell resource center of the Institute of Basic Medical Sciences of Chinese Academy of Medical Sciences. The cells were maintained in Dulbecco’s Modified Eagle’s Medium (DMEM, HyClone, Logan, UT, United States) containing 10% fetal bovine serum (FBS, Gibco, New York, NY, United States) and 1% antibiotic-antimycotic solution (HyClone, Logan, UT, United States). Cell lines were incubated at 37°C in a humidified atmosphere of 5% CO_2_. For the transient transfection assays, the small interfering RNA (siRNA) was purchased from Ruibo Biotech Co., Ltd. (Guangzhou, China). The cells were seeded in 6-well plates and 96-well plates at a density of 4 × 10^5^ and 5 × 10^3^ cells per well, respectively. Then, 5 nM siRNA was transfected by using Lipofectamine 2000 (Invitrogen, Grand Island, NY, United States) according to the manufacturer’s instructions. After transfection for 6 h, the culture medium was replaced, and the cells were incubated with or without various concentrations of compounds for 48 h.

### Cell Viability Assay

A549 and A549/DDP cells were seeded in 96-well plates (Corning, NY, United States) at a density of 5 × 10^3^ cells per well and allowed to attach overnight. The cells or siRNA-transfected cells were treated with various concentrations of compounds at 37°C in 5% CO_2_. After 48 h of exposure, the cells were incubated with 20 μL of 5 mg/mL MTT containing PBS for 4 h. Finally, the MTT containing media was removed, and the insoluble purple formazan crystals produced by live cells were dissolved in 150 μL of DMSO. The plate was placed on a rocking shaker for 10 min, and the optical density of the produced stain was monitored at 570 nm by using a spectrophotometer (Thermo Fisher, Boston, MA, United States).

### Cell Apoptosis Analysis

Cells were seeded in 6-well plates at a density of 5 × 10^5^ cells per well and allowed to attach overnight. The cells or siRNA-transfected cells with the different treatments were incubated at 37°C in 5% CO_2_. After 48 h of treatment, the cells were detached with trypsin and washed twice with cooled PBS. The cells were then treated with Annexin V-FITC followed by treatment with PI for 15 min at 25°C in the dark following the manufacturer’s protocol. Finally, the cell apoptosis status was determined by EPICS XL (Beckman Coulter, United States).

### Colony Formation Assay

Cells and siRNA-transfected cells were treated with different compounds at 37°C in 5% CO_2_. After 48 h of treatment, the cells were seeded in 6-well plates at a density of 5000 cells per well and were incubated for 10 days for the colony formation assay. The cells were fixed with 4% formaldehyde and stained with crystal violet for 10 min.

### RNA Isolation and Quantitative Real-Time PCR (qRT-PCR) Analysis

Total RNA from cultured cells was isolated using TRIzol reagent (TaKaRa Biotech, Kyoto, Japan) according to the manufacturer’s protocol. Total RNA (1 μg) was reverse transcribed to cDNA using a PrimeScript^TM^ RT Reagent Kit with gDNA eraser (TaKaRa Biotech) according to the manufacturer’s instructions. Real-time PCR was performed using the SYBR Premix Ex-Taq^TM^ II Kit (TaKaRa Biotech) in a 7500 real-time PCR System (Applied Biosystems, Foster City, CA, United States), and fold changes were calculated using the 2^-ΔΔ*C*_T_^ method.

### Western Blot Analysis

Protein extracts from cultured cells were prepared by using RIPA lysis buffer containing 1% 100 mM phenylmethanesulfonyl fluoride (Biocolor, Shanghai, China) according to the manufacturer’s specifications, and the protein concentration was determined by the BCA protein assay kit (Thermo Scientific, Rockford, IL, United States). Protein extracts (25 μg) were electrophoresed on a 10% sodium dodecyl sulfate polyacrylamide gel and then transferred onto polyvinylidene fluoride (PVDF) membranes (Millipore, Burlington, MA, United States) at 250 mA for 60 min. After blocking with 5% non-fat milk in TBST (TBS-1% Tween20) for 1 h, the PVDF membranes were incubated overnight at 4°C with anti-GAPDH (1:1000 dilution), anti-G6PD (1:1000 dilution), anti-PGD (1:1000 dilution), anti-TKT (1:1000 dilution), and anti-TALDO1 (1:1000 dilution); next, the membranes were incubated with a rabbit secondary horseradish peroxidase conjugated antibody (1:1000 dilution) for 1 h at room temperature. After that the protein bands were probed with an electro-chemiluminescence Kit (Engreen Biosystem, Beijing, China), and the intensity of protein bands was quantitated by ImageJ software.

### Glucose-6-Phosphate Dehydrogenase Activity Measurement

Cells were seeded in 6-well plates at a density of 1 × 10^6^ cells per well and were allowed to attach overnight. The following day, the cells were transfected with siRNA or treated with 6-AN. After 48 h of treatment, the cells were collected after gently scraping, and the G6PD activity was tested by using a Glucose-6-Phosphate Dehydrogenase Activity Colorimetric Assay Kit (Biovision, Milpitas, CA, United States) according the manufacturer’s specifications. The optical density of the produced stain was monitored at 450 nm by using a spectrophotometer (Thermo Fisher, Boston, MA, United States), and G6PDH activity was calculated according to the manufacturer’s instructions.

### Reactive Oxygen Species (ROS) Measurement

Cells were seeded in 6-well plates at a density of 1 × 10^6^ cells per well and allowed to attach overnight. The following day, the cells were transfected with siRNA or treated with 6-AN. After 48 h of treatment, the culture medium was replaced, and the cells were incubated with 1 mL 10 μM 2,7-dichlorofuorescin diacetate (DCFH-DA, Nanjing Jiancheng Bioengineering Institute, Nanjing, China) containing DMEM for 1 h. Then, the cells were collected with trypsin and washed twice with cooled PBS. Finally, the fluorescence intensity was detected by using a Flex station 3 (Molecular Devices, Sunnyvale, CA, United States).

### NADPH Measurement

6-AN-treated cells or siRNA-transfected cells were collected by gentle scraping; NADP and NADPH were extracted by acid-extracting solution and alkaline-extracting solution, respectively; and the concentrations of NADP and NADPH were tested according to the specifications of a NADP(H) assay kit (Nanjing Jiancheng Bioengineering Institute).

### GSH Measurement

6-AN-treated cells or siRNA-transfected cells were collected by gentle scraping, and the concentrations of GSH and GSSG were extracted and tested by using a GSH assay kit (Nanjing Jiancheng Bioengineering Institute) according to the manufacturer’s specification.

### Metabolomics Assay

A549 cells and A549/DDP cells were harvested, and the large polar metabolites were obtained from lysed cells using an extraction method with an ice-cold methanol/water (4/1) system. Raw data from UPLC-QTOF-MS was pretreated by peak finding, alignment, filtering and normalization to obtain peak data in MarkerView software (AB SCIEX). The metabolomics of A549 and A549/DDP cells was analyzed by using principal component analysis (PCA) and partial least squares discrimination analysis (PLS-DA) in SIMCA-P14.0 software. Differential metabolites were selectively obtained according to the Variable Important in Projection (VIP) value, the confidence interval of the VIP value and the loading plot.

### Statistical Analysis

All experiments were performed independently in at least triplicate. All results are presented as the mean ± SD. Two-tailed Student’s *t*-tests and graphs were performed using GraphPad Prism v6.0c software (GraphPad Software, Inc.). A value of *P* < 0.05 was considered statistically significant.

## Results

### Targeted Metabolomics Revealed Differences in the PPP between A549 and A549/DDP Cells

**FIGURE 1:**
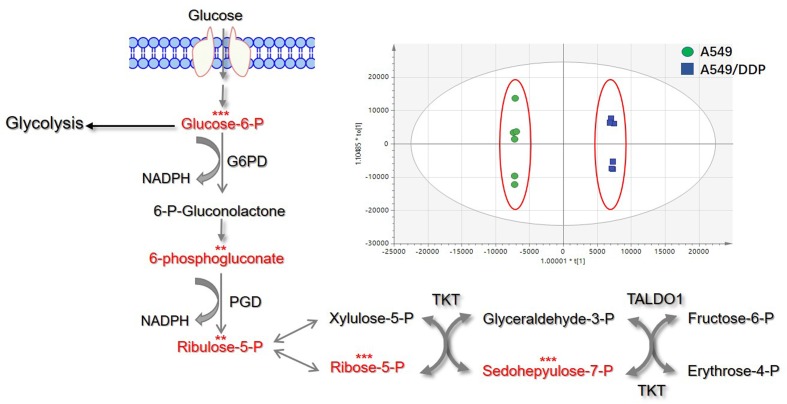
Principal component analysis (PCA) analysis of large polar metabolites and differences in PPP relative metabolites between A549 and A549/DDP cells. The red font represents the differential metabolites. Significant differences are indicated as *^∗^P* < 0.05, *^∗∗^P* < 0.01, and *^∗∗∗^P* < 0.001.

Under principle component analysis, a clear separation of saccharide-related large polar metabolites in A549 and A549/DDP cells was revealed, indicating that distinct metabolite profiles are present between the sensitive and resistant cell lines. Further analysis from targeted metabolomics revealed that multiple metabolites from the PPP in A549/DDP cells, including Glucose-6-phosphate, 6-phosphogluconate, ribulose-5-phosphate, ribose-5-phosphate, sedoheptulose-7-phosphate and glyceraldehyde-3-phosphate, showed significant differences when compared with those in A549 cells, indicating that the metabolic feature of the PPP is significantly different between A549 and A549/DDP cells (**Figure 1**).

### The Redox Status of A549/DDP Cells Was Different from A549 Cells

**FIGURE 2:**
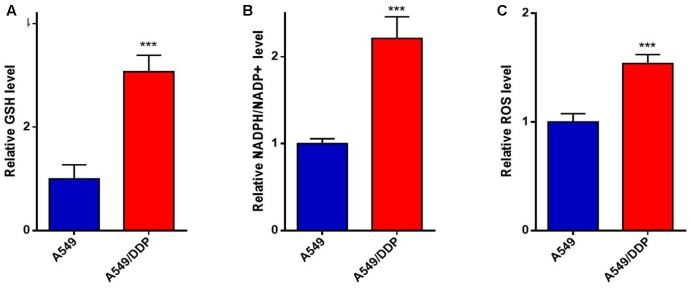
The redox status of A549/DDP cells was different from that of A549 cells. The **(A)** GSH, **(B)** NADPH/NADP+, and **(C)** ROS level were measured in A549 and A549/DDP cells. Data are represented as the means ± SD (*n* = 3), and significant differences are indicated as *^∗^P* < 0.05, *^∗∗^P* < 0.01, and *^∗∗∗^P* < 0.001.

Based on the differences of the PPP metabolites between A549 cells and A549/DDP cells, the variation in the intracellular redox status between the A549/DDP and A549 cell lines was investigated, and the NADPH/NADP+, GSH and ROS levels in A549/DDP cells were significantly higher than those in A549 cells (**Figures 2A–C**).

### The Expression and Activity of G6PD Were Increased in A549/DDP Cells

**FIGURE 3:**
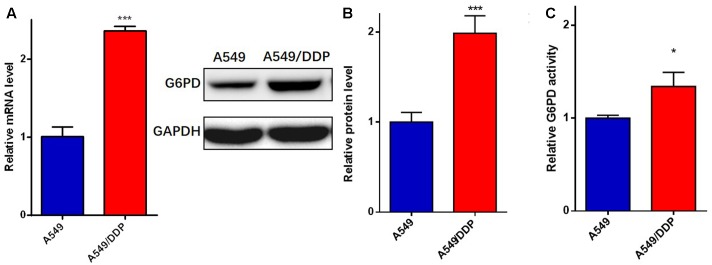
A549/DDP cells displayed higher expression and enzymatic activity of G6PD. The **(A)** mRNA, **(B)** total protein, and **(C)** enzymatic activity of G6PD were assessed in A549 and A549/DDP cells. Data are represented as the means ± SD (*n* = 3), and significant differences are indicated as *^∗^P* < 0.05, *^∗∗^P* < 0.01, and *^∗∗∗^P* < 0.001.

Due to the differences in the redox status and PPP metabolism between A549 and A549/DDP cells, considering that the oxidative branch maintains the homeostasis of the distinct redox system, we hypothesized that the oxidative PPP activity of A549/DDP cells was better than that of A549 cells. As a major checkpoint for the activity of the oxidative PPP, the mRNA, protein level and activity of G6PD were relatively higher in A549/DDP cells (**Figures 3A–C**) than in A549 cells.

### Inhibition of G6PD Restored the Sensitivity of A549/DDP Cells to Cisplatin

**FIGURE 4:**
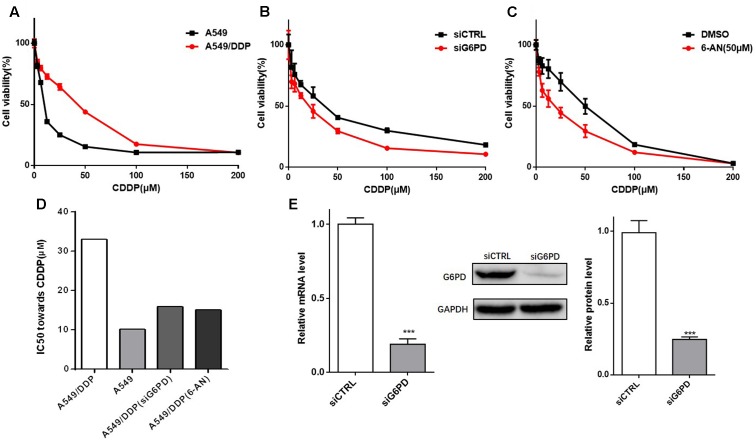
Inhibition of G6PD significantly decreased the cell viability of A549/DDP cells to cisplatin. **(A)** The resistance of A549/DDP cells was determined by a MTT assay. **(B)** A549/DDP cells transfected with siRNA were cultured for 48 h with various concentrations of cisplatin (3.125– 200 μM), and the cell viability was determined. **(C)** A549/DDP cells were treated with cisplatin alone or in combination with 6-AN for 48 h, and cell viability was determined. **(D)** The IC_50_ toward CDDP in **(A–C)** were calculated and is presented as a column chart. **(E)** The reduction of G6PD expression by siRNA was confirmed by qRT-PCR and western blot analysis. Data are presented as the means ± SD (*n* = 3), and significant differences are indicated as *^∗^P* < 0.05, *^∗∗^P* < 0.01, and *^∗∗∗^P* < 0.001.

MTT assay was performed to further determine the drug resistance of the A549/DDP cells to cisplatin. The half-maximal inhibitory concentration (IC50) of A549/DDP cells was significantly higher than that of A549 cells (**Figures 4A,D**), which confirmed that A549/DDP cells were cisplatin resistant.

**FIGURE 5:**
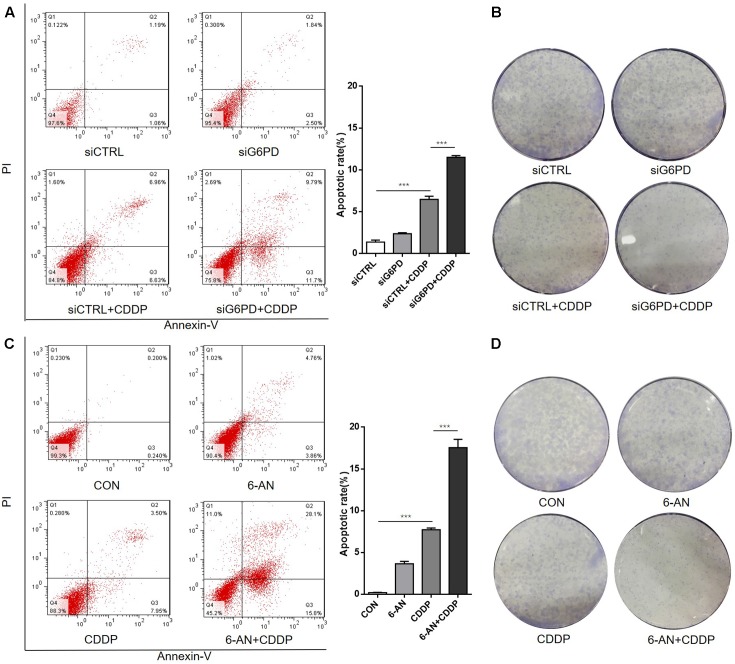
Inhibition of G6PD sensitizes the A549/DDP cells to cisplatin by inducing apoptosis and inhibiting colony formation. **(A)** A549/DDP cells transfected with siRNA were cultured with or without cisplatin for 48 h followed by staining with Annexin-V/PI. The lower right quadrant (Annexin-V+/PI–) indicates the percentage of early apoptosis. **(B)** The cells were stained with crystal violet for colony formation after culturing for an additional 10 days. **(C)** A549/DDP cells were treated with cisplatin and 6-AN separately or in combination for 48 h followed by staining with Annexin-V/PI. **(D)** Cells were stained with crystal violet for colony formation after culturing for an additional 10 days. Data are presented as the means ± SD (*n* = 3), and significant differences are indicated as *^∗^P* < 0.05, *^∗∗^P* < 0.01, and *^∗∗∗^P* < 0.001.

To investigate whether the cisplatin sensitivity of A549/DDP cells could be enhanced by inhibition of G6PD, A549/DDP cells transfected with siCTRL or siG6PD were exposed to different concentrations of cisplatin for 48 h, and cell viability was tested (the reduction of G6PD expression by siRNA was presented in **Figure 4E**). The results showed that the si-G6PD group was much more sensitive to cisplatin than the siCTRL group (**Figures 4B,D**). The apoptotic status of the A549/DDP cells transfected with siCTRL or siG6PD, after treating with cisplatin for 48 h, was further determined. The results showed that the apoptotic rate of the siG6PD group was much higher than that of the siCTRL group when exposed to CDDP (**Figure 5A**), and colony formation assays confirmed that transfection with siG6PD could inhibit the ability of A549/DDP cells to form colonies (**Figure 5B**). These findings indicated that knockdown of G6PD facilitates the efficacy of cisplatin in A549/DDP cells.

Additionally, G6PD inhibitor 6-AN was used to further evaluate the role of G6PD on the sensitivity of A549/DDP cells. Similar to the results of transfecting siG6PD, the cell viability and the IC_50_ of A549/DDP cells co-treated with 6-AN and cisplatin effectively decreased (**Figures 4C,D**), and the apoptotic rate of the co-treatment group was much higher than that of the mono-treatment group (**Figure 5C**). The colony formation ability of the co-treatment group was also significantly lower than that of mono-treatment group (**Figure 5D**). These results indicated that inhibition of G6PD activity enhanced the sensitivity of cisplatin in A549/DDP cells.

### Inhibition of G6PD Sensitized A549/DDP Cells to Cisplatin through Inducing Oxidative Stress

**FIGURE 6:**
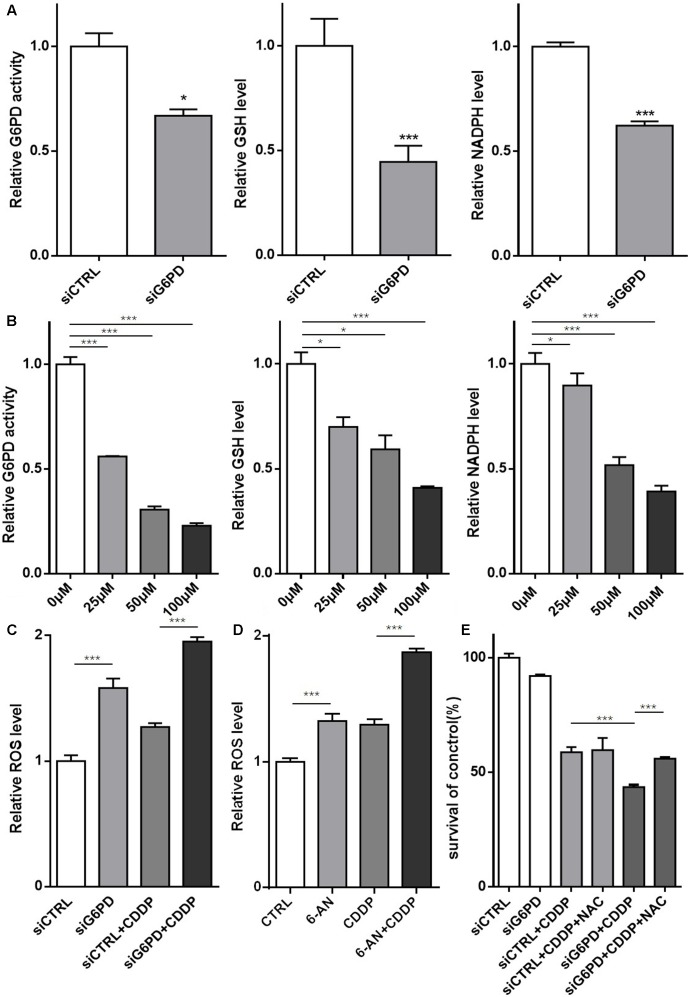
Inhibition of G6PD sensitized A549/DDP cells to cisplatin through inducing oxidative stress. **(A)** The relative G6PD enzymatic activity and the GSH and NADPH levels were assessed in A549/DDP cells transfected with siRNA. **(B)** The relative G6PD enzymatic activity and GSH and NADPH levels were assessed in A549/DDP cells treated with various concentrations of 6-AN. **(C)** A549/DDP cells transfected with siRNA were treated with or without cisplatin for 48 h, and the ROS level was assessed. **(D)** A549/DDP cells were treated with cisplatin and 6-AN separately or in combination for 48 h, and the ROS level was assessed. **(E)** A549/DDP cells transfected with siRNA were treated with cisplatin and NAC separately or in combination for 48 h, and the cell viability was assessed by MTT assay. Data are presented as the means ± SD (*n* = 3), and the significant differences are indicated as *^∗^P* < 0.05, *^∗∗^P* < 0.01, and *^∗∗∗^P* < 0.001.

Since there is an intimate connection between G6PD and redox homeostasis, and it is well established that cisplatin treatment could induce ROS accumulation, thereby disrupting intracellular redox system in cancer cells, we hypothesized that inhibition of G6PD sensitizes A549/DDP cells to cisplatin through blocking the antioxidant ability of A549/DDP cells, finally weakening the resistance. Based on these findings, the G6PD activity and GSH, NADPH and ROS levels were tested, and the results showed that regardless of transfection with siG6PD or treatment with 6-AN, the G6PD activity and the GSH and NADPH levels were significantly decreased, and the ROS level was elevated in A549/DDP cells (**Figures 6A–D**). In addition, compared with non-depletion, G6PD depletion effectively induced more ROS accumulation after cisplatin exposure (**Figures 6C,D**). To further confirm our hypothesis, we used the antioxidant NAC, and we discovered that NAC could rescue the decreased viability of A549/DDP cells when transfected with siG6PD followed by treating with cisplatin (**Figure 6E**). In summary, inhibition of G6PD sensitized A549/DDP cells to cisplatin through inducing intracellular oxidative stress, which caused by GSH and NADPH depletion and ROS accumulation.

### Other Major Enzymes of the PPP Did Not Participate in the Cisplatin Resistance of A549/DDP Cells

**FIGURE 7:**
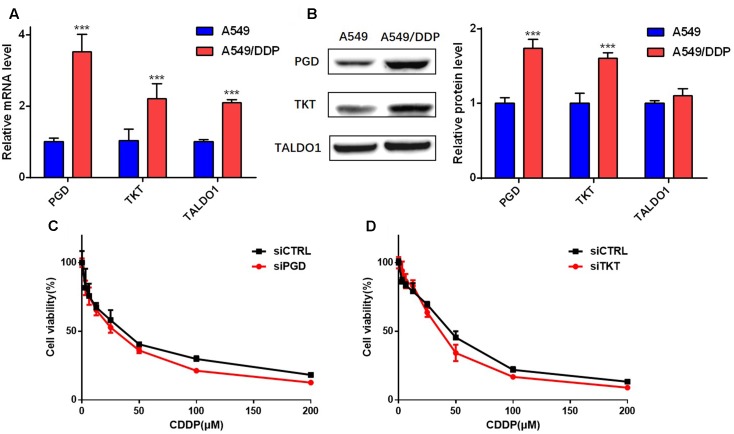
Other major enzymes of the PPP did not participate in the cisplatin resistance of A549/DDP cells. **(A,B)** The mRNA and total protein levels of PGD, TKT and TALDO1 were assessed in A549 and A549/DDP cells. The control GAPDH band is shown in **Figure 3B**. **(C,D)** A549/DDP cells transfected with siRNA were cultured for 48 h with various concentrations of cisplatin (3.125–200 μM), and cell viability was determined. Data are represented as the means ± SD (*n* = 3), and significant differences are indicated as *^∗^P* < 0.05, *^∗∗^P* < 0.01, and *^∗∗∗^P* < 0.001.

In addition to G6PD, PGD is another key enzyme of the oxidative PPP branch that could generate NADPH. In our study, similar to G6PD, the expression level of PGD in A549/DDP cells was also relatively higher than that in A549 cells, and the effect of the knockdown of PGD was also investigated in the A549/DDP cells (**Figures 7A,B**). Unfortunately, inhibition of PGD by siRNA exhibited no remarkable improvement in the drug sensitivity of A549/DDP cells (**Figure 7C**). In addition, in order to determine the specific role of the oxidative PPP in the cisplatin-induced resistance of A549/DDP cells, we tested the corresponding parts of the non-oxidative PPP enzyme TKT and TALDO1. Similar to G6PD and PGD, A549/DDP cells also expressed relatively high levels of TKT but not TALDO1 (**Figures 7A,B**), and knockdown of TKT also showed no significant effects on the resistance of A549/DDP cells (**Figure 7D**). The above results indicated that other enzymes of the PPP had little effect on the cisplatin resistance of A549/DDP cells in our experiments.

## Discussion

Cisplatin is widely applied in the clinical treatment for various cancers, including bladder, ovarian, colorectal, lung and head and neck cancers. However, the acquired resistance toward cisplatin remains a vital problem that is too complicated to fully solve (Amable, 2016). Therefore, numerous efforts have been made to work on this problem. The Warburg effect is regarded as the most prominent characteristic in cancers, and accumulating evidence suggests that such a hallmark is essential for a resistant phenotype in cancer cells (Morandi and Indraccolo, 2017). Recently, increasing attention has been put on the PPP, which produces ribose and NADPH through the non-oxidative branch and oxidative branch, respectively. Targeting the PPP in tumors is an effective approach to control cancer development and promote therapeutic efficacy. In this study, we found that the PPP metabolites of the cisplatin-induced resistant A549/DDP cells were distinctive from its parental A549 cells, which indicated that there might be some correlations between the PPP and the resistance of A549/DDP cells to cisplatin.

Cancer cells demand high ROS concentrations to maintain their accelerated metabolism and high proliferation rate (Sosa et al., 2013). To avoid large amounts of ROS accumulation, they may experience a process of Redox Resetting, thus obtaining a new redox system (Liu et al., 2016). Therefore, variation in the redox status is considered an important factor in the development of chemoresistance. The nuclear factor erythroid-related factor 2 (Nrf2) is a transcription factor that plays a significant role in protection against oxidative stress to maintain intrinsic redox homeostasis, and numerous studies suggest that the cellular protection provided by an abnormal expression of Nrf2 confers cancer cells a proliferation advantage and the ability to resist antitumor drugs (Samatiwat et al., 2016; Bai et al., 2017; Roh et al., 2017). In our previous study, we found that the expression of Nrf2 in A549 cell was lower than that in A549/DDP cells, and the transfection of A549/DDP cells with Nrf2 siRNA to suppress the Nrf2 signaling pathway restored the susceptibility of A549/DDP cells to cisplatin (Xia et al., 2015). Additionally, it was recently revealed that Nrf2 could directly activate genes in the PPP [G6PD, PGD, TKT, and transaldolase 1 (TALDO1)] (Mitsuishi et al., 2012; Singh et al., 2013). In this study, we found differences in the redox status between A549 and A549/DDP cells. The results indicated that the resistance of A549/DDP cells might be reversed through regulating the redox homeostasis via the PPP.

Due to the generation of NADPH, cancer cells with hyper PPP activity always possess a different intracellular redox system, which is more insusceptible to oxidative stress induced by chemotherapeutic agents. G6PD catalyzes the production of the first molecule of NADPH and harmonizes the two branches of the PPP. Some studies have found that G6PD presented high activities in some cancers, while others put forward the hypothesis of a lower cancer incidence in G6PD deficient subjects (Cohen et al., 1968; Cocco, 1987). G6PD inhibitor was further revealed to exert anti-proliferation and cytotoxic effect on cancer cells. In recent years, more and more research groups have paid attention to the important role of G6PD in various cancer cells, including colorectal cancer, cervical cancer, renal cell carcinoma, esophageal squamous cell carcinoma, lung cancer and head and neck cancer (Di et al., 1997; Lucarelli et al., 2014; Catanzaro et al., 2015; Fang et al., 2016; Wang et al., 2016; Ju et al., 2017; Zhang et al., 2017). Cisplatin takes effect by damaging nuclear DNA, followed by inducing ROS accumulation and increasing oxidative stress, finally killing cancer cells. The elevated expression and activity of G6PD generates high levels of NADPH to reduce ROS-induced oxidative stress and leads to a higher production of ribose for DNA synthesis and repair, thereby influencing the efficacy of cisplatin (Patra and Hay, 2014). In addition, GSH is also a major contributing factor to cisplatin resistance by binding to or reacting with cisplatin (Traverso et al., 2013). Inhibition of G6PD restores cisplatin sensitivity in ovarian cancer cells and enhances the sensitivity of colorectal cancer cells to oxaliplatin treatment (Catanzaro et al., 2015; Ju et al., 2017). In our study, we found a relatively high expression and enzymatic activity of G6PD in the A549/DDP cells and put forward that the G6PD-mediated intracellular redox homeostasis was associated with the cisplatin effects in NSCLC cisplatin-induced resistant A549/DDP cells.

To confirm our hypothesis, a siRNA and an inhibitor were used to investigate the role of G6PD in cisplatin-induced chemoresistance. We found that the attenuation of G6PD resulted in a decreased IC50 in A549/DDP cells, both by reducing proliferation and promoting apoptosis after cisplatin exposure. Besides, it was discovered that inhibition of G6PD with the siRNA or 6-AN showed no obvious effects on cell apoptosis or colony formation, which suggested that cisplatin resistant A549 cells could not be suppressed well just by inhibiting G6PD, and this may be caused by its high proliferating rate. A previous study suggested that G6PD inhibitor 6-AN sensitized various cancer cells to cisplatin through increasing cisplatin accumulation and Pt-DNA adduct formation (Budihardjo et al., 1998), but whether the inhibition of G6PD sensitized cisplatin via regulating the PPP activity and redox homeostasis remained unclear. Our results showed that the inhibition of G6PD by siRNA or an inhibitor could directly disrupt the redox homeostasis, which was well presented by the GSH and NADPH depletion and ROS accumulation. In addition, treatment with the mechanistical antioxidant NAC rescued the drug resistance of A549/DDP cells transfected with siG6PD, which further confirmed that inhibition of G6PD rescued the cisplatin resistance of A549/DDP cells through affecting its redox status.

It has been reported that redox homeostasis is required for the survival of cisplatin-resistant ovarian cancer cells (Catanzaro et al., 2015) and recently, another study revealed that disrupting G6PD-mediated redox homeostasis enhances the chemosensitivity of oxaliplatin in colorectal cancer, which is consistent with our study in lung cancer (Ju et al., 2017). These studies indicated that affecting redox status by inhibiting G6PD could be a valid way to overcome chemoresistance. But whether other enzymes from the PPP involving redox would affect the resistance remains unknown. To determine the specific role that G6PD plays in the cisplatin-induced resistance of A549/DDP cells, we investigated the expression level of PGD, TKT and TALDO1 and found a significant higher mRNA and protein level of PGD and TKT in the A549/DDP cells. Interestingly, the discrepancy of mRNA and protein level of TALDO1 was not consistent in our experiments, and whether that is associated with the resistance requires further research. Besides, knockdown of PGD and TKT by siRNA showed no obvious effects on cisplatin resistance. Therefore, we considered that G6PD was a specific enzyme that is important for the cisplatin resistance of A549/DDP cells.

In summary, our data indicated that G6PD provided an intimate link between the redox status and cisplatin resistance through activating the PPP, and it also showed new evidence that the inhibition of G6PD rendered A549/DDP cells more susceptible to cisplatin. Further study is needed to investigate the role of G6PD in chemoresistance *in vivo*. Whether the regulators upstream of the PPP have effects on cisplatin resistance in cancers also deserves further research and a better understanding.

## Author Contributions

The experiments carrying out, data analysis, and manuscript writing were conducted by WH, PC, WY, CX, and DC. ZZ and MH provided financial and technical support for the research. JJ founded the research project, designed the experiments, writing the manuscript, proofread the manuscript and contributed to the funding of the project.

## Conflict of Interest Statement

The authors declare that the research was conducted in the absence of any commercial or financial relationships that could be construed as a potential conflict of interest.
